# Impact of Thermosonication Treatment on Parsley Juice: Particle Swarm Algorithm (PSO), Multiple Linear Regression (MLR), and Response Surface Methodology (RSM)

**DOI:** 10.1021/acsomega.4c02749

**Published:** 2024-06-26

**Authors:** Dilek Dulger Altıner, Seydi Yıkmış, Mehmet Ali Şimşek, Melikenur Türkol, Nazan Tokatlı Demirok, Guler Celik

**Affiliations:** † Department of Gastronomy and Culinary Arts, Tourism Faculty, Kocaeli University, 41400 Kartepe, Kocaeli, Türkiye; ‡ Department of Food Technology, Tekirdag Namık Kemal University, 59830 Tekirdag, Türkiye; § Department of Computer Technologies, Vocational School of Technical Sciences, Tekirdag Namik Kemal University, 59030 Tekirdag, Türkiye; ∥ Nutrition and Dietetics, Faculty of Health Sciences, Halic University, 34060 Istanbul, Türkiye; ⊥ Department of Nutrition and Dietetics, Faculty of Health Sciences, Tekirdag Namık Kemal University, 59030 Tekirdag, Türkiye; # The Scientific and Technological Research Council of Turkey, Bursa Test and Analysis Laboratory (TUBITAK BUTAL), Bursa 16190, Türkiye

## Abstract

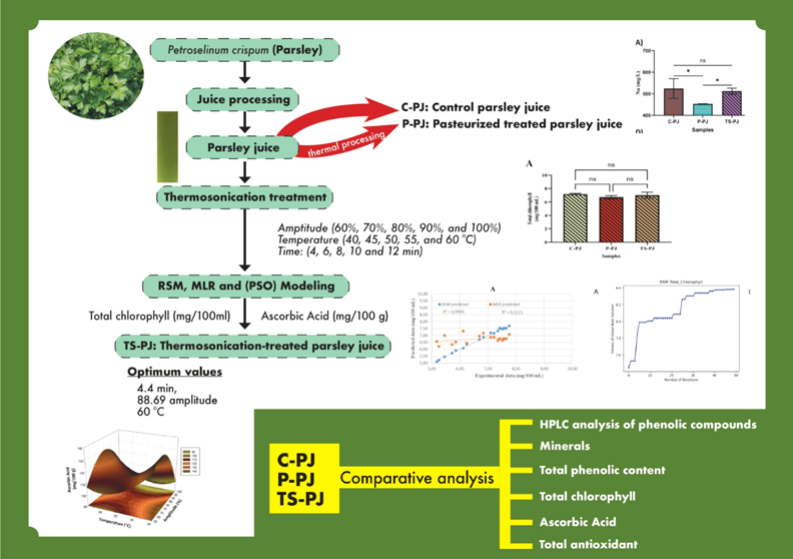

Thermosonication (TS), also known as ultrasonic-assisted heat treatment, is gaining attention in liquid product processing due to its ability to improve quality parameters and can serve as an alternative to thermal treatments. The parsley juice (TS-PJ) was subjected to thermosonication treatment (frequency: 26 kHz; power: 200 W; amplitude 60, 70, 80, 90, and 100%; temperature: 40, 45, 50, 55, and 60 °C; time: 4, 6, 8, 10, and 12 min) and was compared with untreated control parsley juice (C-PJ) and pasteurized treated (P-PJ) (85 °C/2 min) parsley juice samples. The objectives of the research work were to determine the effect of thermosonication on the quality attributes such as total chlorophyll and ascorbic acid of parsley juice using particle swarm algorithm (PSO), multiple linear regression (MLR), and response surface methodology (RSM). Thermosonication enhanced the bioactive compounds of parsley juice. The results showed that 15 phenolic compounds were detected in the samples. There was a significant (*p* < 0.05) increase in gallic acid contents in ultrasound-treated TS-PJ. There was no significant difference in total chlorophyll and ascorbic acid content between C-PJ and TS-PJ samples. Na and K from macro minerals and Fe and Zn from micro minerals were high in PJ samples. While K contents were increased, P contents were lower in the TS-PJ sample. RSM modeling provided superior prediction compared to MLR. PSO, on the other hand, made good predictions intuitively. Thermosonication enriched parsley juice’s bioactive components and had positive health effects.

## 1 Introduction

*Petroselinum crispum* (Parsley) belongs to the family Apiaceae, which is used to enhance the aroma, flavor, and color of foods.^1^*P. crispum* has drawn researchers’ attention to its significant bioactive compounds, such as phenolic acids. Due to these contents, *P. crispum* has a strong antioxidant effect, too.^2^ It has hepatoprotective, analgesic, anti-inflammatory, antidiabetic, antibacterial, and antifungal activities.^3^

Vegetables are essential human diet components with bioactive compounds, such as phenolics, flavonoids, carotenoids, vitamins, and minerals. Vegetable juices are a functional way to intake these plant ingredients.^4^ Microbial spores and pathogen inhibition is necessary to ensure juice’s safety.^5,6^ Traditional methods, such as thermal processing, have decreased food contamination. However, heat can also cause a reduction of the organoleptic properties and nutritional value of foods. Ultrasound technology is a potential alternative to traditional thermal pasteurization technology and promising nonthermal processing.^7^ Ultrasound is an eco-friendly and emerging sustainable technology widely researched in food preservation and processing.^8−11^ Furthermore, ultrasound technology is sustainable, simple, and economical.^12^ Ultrasound treatment changes the particle size of vegetable juice (particulate solid−liquid systems).^13^

Response surface methodology (RSM) is a statistical and mathematical method for optimizing fast, low-cost random processes.^14,15^ Thanks to RSM, we understand the interaction between the effects and the factors, parameters, and equations that relate to experimental parameters and responses.^16−18^ Particle swarm optimization (PSO) algorithm was proposed by Kennedy and Eberhart.^19^ PSO is a population-based meta-heuristic optimization algorithm motivated by the cooperative behavior of foraging animals such as flocks of birds or schools of fish.^19,20^ Multiple linear regression (MLR) is a statistical analysis method used in machine learning. Few studies are using these methods for juice optimization.^21−25^ The combination of RSM, MLR, and PSO can provide more accurate results in component optimization in vegetable juices. When the literature was scanned, no studies were found investigating the optimization of parsley juice’s chlorophyll and ascorbic acid components with RSM, MLR, and PSO after ultrasound application. The main aim of this study was to determine the impact of ultrasound technology on the antioxidant activity, bioactive ingredients, phenolic compounds, and mineral contents of parsley juice using the particle swarm algorithm (PSO), response surface methodology (RSM), and multiple linear regression (MLR) optimization. At the same time, the levels of these components after pasteurization were investigated. This study will also optimize total chlorophyll and ascorbic acid equations (formulas) obtained from the RSM and MLR.

## 2 Materials and Methods

### 2.1 Materials

Parsley juice samples were collected from local producers (Tekirdag, Turkiye) and kept at 4 °C until the experiments were performed. Stems and ripened parts were discarded. Parsley was crushed by using a blender (Waring Commercial Blender Model HGB2WTS3). The sample was then filtered, mixed with a vortex, and selected as the control (CP-J).

### 2.2 Methods

#### 2.2.1 Thermal Pasteurization Treatment

The produced samples of parsley juice were transferred into 100 mL glass containers and subjected to pasteurization treatment at 85 ± 1 °C for 2 min. This pasteurization used a water bath apparatus (Wisd Model WUC-D06H, Daihan, Wonju, Korea). Subsequently, pasteurized parsley juice (P-PJ) samples were cooled to 20 ± 1 °C at room temperature and stored at −20 ± 1 °C until needed for further analytical assessment.

#### 2.2.2 Thermosonication Treatment

For thermosonication treatment (TS), 100 mL of P-PJ was processed using a ultrasonic processor (Hielscher Ultrasonics Model UP200 St, Berlin, Germany) at a frequency of 26 kHz and 200 W. Ultrasound parameters studied included amplitude (60, 70, 80, 90, and 100%), processing duration (4, 6, 8, 10, and 12 min), and temperature (40, 45, 50, 55, and 60 °C) in constant mode. An ice−water bath was used to prevent overheating during ultrasound processing. After TS, the samples (TS-PJ) were immediately cooled in an ice bath and were kept at −18 ± 1 °C until analysis.

#### 2.2.3 Response Surface Methodology (RSM)

Response surface methodology (RSM) is a statistical technique used to examine the relationship between explanatory variables and response variables.^26^ RSM and MLR were used to understand the effect of the thermosonication process on chlorophyll and ascorbic acid in parsley juice. For thermosonication, time (*X*_1_, 4−12 min), amplitude (*X*_2_, 60−100%), and temperature (*X*_3_, 40−60 °C) are independent factors, while total chlorophyll (mg/100 mL) and ascorbic acid (mg/100 g) are response variables. Central composite design (CCD) was implemented using Minitab software (Version 19, Minitab software, State College, PA) to optimize the thermosonication process of parsley juice for RSM. The number of experiments obtained from the 3-factor CCD is 20 (Table 1). Each experiment was performed in triplicate.

**Table 1:** Chlorophyll and Ascorbic Acid Results with Dependent and Independent Variables of RSM and MLR Analysis of Thermosonication^a^

	**independent variables**			**dependent variables**					
				**total chlorophyll(mg/100 mL)**			**ascorbic acid(mg/100 g)**		
run no.^**a**^	**time (*X***_**1**_**)**	**amplitude (*X***_**2**_**)**	**temperature (*X***_**3**_**)**	**experimental data**	**RSM predicted**	**MLR predicted**	**experimental data**	**RSM predicted**	**MLR predicted**
**1**	6	70	45	7.76 ± 0.04	7.69	7.07	128.16 ± 2.83	126.93	126.59
**2**	8	80	50	7.41 ± 0.10	7.53	6.76	114.86 ± 1.62	116.17	113.52
**3**	6	90	45	5.86 ± 0.08	5.90	6.52	113.32 ± 1.60	112.41	119.65
**4**	10	90	45	5.72 ± 0.54	5.71	6.34	105.30 ± 1.15	104.14	107.19
**5**	6	70	55	6.84 ± 0.48	6.88	7.18	123.12 ± 2.81	124.51	119.85
**6**	10	70	55	5.46 ± 0.41	5.45	7.00	98.28 ± 1.67	99.42	107.39
**7**	6	90	55	7.52 ± 0.53	7.47	6.63	124.50 ± 2.12	123.32	112.91
**8**	8	80	50	7.58 ± 0.11	7.53	6.76	116.47 ± 1.65	116.17	113.52
**9**	8	80	50	7.56 ± 0.13	7.53	6.76	115.92 ± 1.36	116.17	113.52
**10**	10	90	55	6.25 ± 0.09	6.36	6.45	100.53 ± 1.42	101.98	100.45
**11**	4	80	50	6.70 ± 0.47	6.71	6.94	120.60 ± 1.34	121.70	125.98
**12**	12	80	50	5.18 ± 0.37	5.09	6.58	89.69 ± 0.59	88.34	101.06
**13**	8	60	50	6.12 ± 0.06	6.09	7.31	108.78 ± 2.81	107.59	120.46
**14**	8	100	50	5.26 ± 0.37	5.21	6.21	94.68 ± 1.82	95.62	106.58
**15**	8	80	40	7.35 ± 0.16	7.33	6.65	132.3 ± 2.08	133.30	120.26
**16**	8	80	60	7.22 ± 0.51	7.16	6.87	129.96 ± 2.57	128.72	106.78
**17**	8	80	50	7.44 ± 0.63	7.53	6.76	116.43 ± 0.37	116.17	113.52
**18**	8	80	50	7.65 ± 0.65	7.53	6.76	117.70 ± 1.78	116.17	113.52
**19**	8	80	50	7.65 ± 0.18	7.53	6.76	115.92 ± 1.15	116.17	113.52
**20**	10	70	45	7.10 ± 0.03	7.18	6.88	113.52 ± 1.22	114.92	114.13
**TS-PJ**	4.4	88.69	60	7.79			145.42		
**experimental values**				7.34 ± 0.64			139.23 ± 1.87		
**% difference**				5.78			4.26		

a *X*_1_—time; *X*_2_—amplitude; RSM: response surface methodology; MLR: multiple linear regression TS- PJ: thermosonication-treated parsley juice.

#### 2.2.4 Multiple Linear Regression (MLR)

The relationship between the independent and dependent variables of the data set obtained in the laboratory environment was estimated using multiple linear regression (MLR). The results obtained with MLP are compared with those obtained with RSM.

MLR is a statistical analysis method used in the context of machine learning. It is used to determine the relationship between the dependent variable of a data set and several independent variables. This method can be used to understand the relationships among data and predict future values. The function expresses the MLR model in eq 1. In the equation, *y* is the dependent variable, *X*_1_, *X*_2_, ···, *X*_*n*_ are the *n* independent variables, β_0_ is the error term, and β_1_,β_2_,···,β*_n_* are the regression weight coefficients.
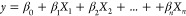
1

To clarify the performance of RSM and MLR models, the determination coefficient (*R*^2^), root-mean-square error (RMSE), and absolute average deviation (AAD) were compared between MLR and RSM models. The formulas are written as follows:
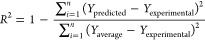
2
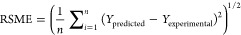
3
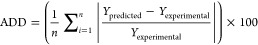
4where *n*, *Y*_average_, *Y*_predicted_, and *Y*_experimental_ are the number of data points, the average of data, the predicted value, and the experimental value, respectively. The accuracy and validity of the model were measured on the basis of *R*^2^, AAD, and RMSE.

#### 2.2.5 Modeling of PSO

**Figure 1:**
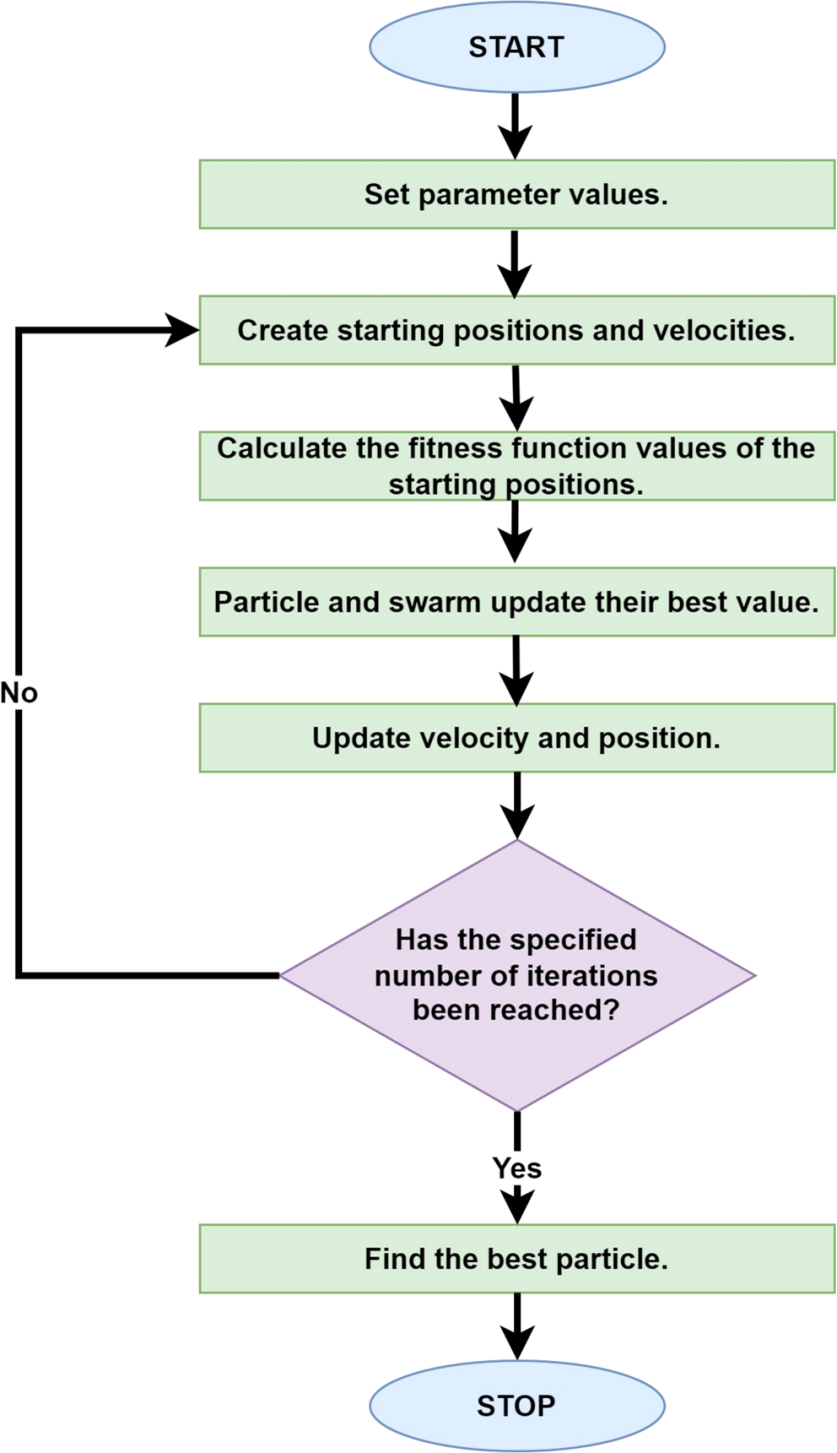
Flowchart of the PSO algorithm.

In the PSO algorithm, each particle represents a possible solution to the problem, and the entirety of the particles is termed a swarm (population). The PSO algorithm begins by determining the parameter values to be used. The initial positions and velocities of each particle in the swarm are determined. The objective function values of the initial positions for the particles are calculated. The best values for the particles and the best values for the swarm have been updated. Speed and position are updated. The most optimal particle is found when the algorithm reaches the number of iterations for termination. If the algorithm has not reached the termination criterion, then the algorithm continues by recalculating the objective function using the new position and velocity information. The flowchart of the PSO algorithm is shown in Figure 1.

In the PSO algorithm, the position of each particle is adjusted according to its own experience and that of the swarm. The particles are randomly placed in the solution space and then move toward the best solution. This is done by each particle following its own best solution (*pBest*) and the best solution in the swarm (*sBest*).

The velocity (*V*) and direction of each particle were optimized and calculated using the velocity formula. This formula contains factors such as the current velocity of the particle (*V*_*ij*_(*t*)), the difference between the best position (*y*_*ij*_^*pBest*^(*t*)) and the current position *x*_*ij*_(*t*) of the particle, and the difference between the best position *y*_*j*_^*sBest*^(*t*) of the entire swarm and the current position *x*_*ij*_(*t*) of the particle. The velocity formula is used to determine the direction in which the particles move in the next step. This allows the algorithm to find the optimal result of the optimized fitness function. The velocity equation is given in eq 5.
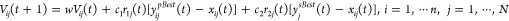
5

In eq 5, *wV*_*ij*_(*t*) is the velocity of particle *i* of size *j* = 1, ···, and *N* at time *t*. *w* is the inertia weight. The particle needs to maintain its former speed and therefore needs a coefficient of inertia. *x*_*ij*_(*t*) is the position of the particle *i* in dimension *j* at time *t*. *c*_1_ is the cognitive coefficient. *c*_2_ is the social coefficient. *r*_1*j*_ and *r*_2*j*_ are random numbers generated from a uniform distribution in the interval (0,1). The values *r*_1*j*_ and *r*_2*j*_ add a stochastic property to the solution at time *t*. The new position of particle *i* at time *t* + 1 is determined by adding the velocity to its current position. The equation for the new position information is shown in eq 6.
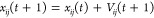
6

The new position of particle *i* at time *t* + 1 was measured by how close the solution in the fitness function *f* is to the optimum. The particle’s best position and the swarm’s best position are updated. The equation for updating the best position of the particle is shown in eq 7. The equation is formulated as a maximization problem. If the solution generated from the fitness function is greater than the best solution of the particle, it is updated as the new best value.
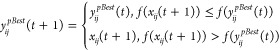
7

The equation that compares the new position of particle *i* at time *t* + 1 with the best position information found by all particles in the swarm is given in eq 8. Thus, the best position information found by the swarm is also updated.
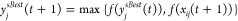
8

#### 2.2.6 Total Chlorophyll

Chlorophyll was estimated according to the method described by Hiscox and Israelstam.^27^ 3 mL of parsley juice was mixed with 3 mL of acetone (80% v/v) and then filtered three times using Whatman filter paper. The absorbance of the resulting filtrate was measured at 645 and 663 nm. The total chlorophyll content was calculated using the following equations:
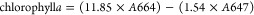
9
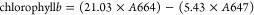
10
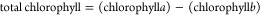
11

#### 2.2.7 Ascorbic Acid Content

Ascorbic acid content was measured by Ordóñez-Santos and Vázquez-Riascos.^28^ Thirty milliliters of parsley juice were mixed with 0.2 g of C_2_H_2_O_4_. Then, 10 mL of the solution was titrated with 2,6-dichloroindophenol (DPIP) reagent until a permanent dark purplish-red color was achieved. The concentration of ascorbic acid was determined using eq 12.
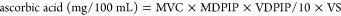
12

MVC is the molar mass (g/mol) of ascorbic acid; CDPIP is the molar concentration (mol/L) of 2,6-dichloroindophenol (DPIP); VS is the sample volume (L); and VDPIP is the volume of DPIP (L).

#### 2.2.8 Total Phenolic Compounds (TPC)

The total phenolic content (TPC) was quantified using the Folin-Ciocalteau method.^29^ For the TPC analysis of strawberry vinegar samples, 2 mL of each was combined with 8 mL of 80% methanol, followed by centrifugation at 4000 rpm for 20 min, assuming a dilution factor 5 (calculated as 10/2). Subsequently, 50 μL of the supernatant was transferred into a glass tube, followed by addition of 100 μL of Folin-Ciocalteu reagent and 1500 μL of deionized water. The mixture was allowed to stand for 10 min. After this incubation, 50 μL of a 20% sodium carbonate (Na_2_CO_3_) solution was added. The mixture was then left to react in the dark for 2 h. The absorbance of the strawberry vinegar samples was measured at 765 nm using a blank for calibration. The results were expressed in milligrams of gallic acid equivalents per 100 mL of the sample.

#### 2.2.9 Antioxidant Activity

The antioxidant method determined the DPPH activity, which uses the DPPH (2,2-diphenyl-1-picrylhydrazyl) radical based on inhibition with some modifications.^30^ First, 2.9 mL of 0.1 mM DPPH solution (prepared in ethanol) was added to 0.1 mL of the fruit juice sample, mixed by vortexing, and allowed to stand in the dark for 30 min at room temperature. The absorbance was then measured on a UV−vis spectrophotometer (SP-UV/vis-300SRB, Australia). The wavelength used was 517 nm. The scavenging activity of the DPPH radicals was calculated as follows. The calculation was made using eq 13.
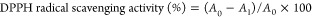
13where *A*_0_ is the absorbance of the control and *A*_1_ is the absorbance of the juice.

#### 2.2.10 Analysis of Phenolic Compounds

The detection of phenolic compounds was analyzed using an Agilent 1260 Infinity chromatograph with a diode array detector (DAD). That procedure was as described by Portu et al.^31^ The flow rate was 0.80 mL/min, and the column temperature was fixed at 30 °C. Solution A and B consisted of water with phosphoric acid (0.1%) and acetonitrile, respectively. The adopted elution gradient was applied as follows: 17% B (0 min), 15% (7 min), 20% (20 min), 24% (25 min), 30% (28 min), 40% (30 min), 50% (32 min), 70% (36 min), and 17% (40 min). Phenolic ingredients were determined according to the UV−vis data obtained from authentic standards and the retention times of the available pure compounds. Chromatograms were registered at 280, 320, and 360 nm. The findings were expressed as μg/mL.

#### 2.2.11 Analysis of Minerals

The mineral composition of the parsley juice was determined using a method previously expressed by Sezer et al. (2019).^32^ For the samples, calcium (Ca), copper (Cu), iron (Fe), manganese (Mn), magnesium (Mg), sodium (Na), potassium (K), copper (Cu), and zinc (Zn) content analyses were performed with an inductively coupled plasma optical emission spectrometer (PerkinElmer 2100 Dual View). Analytical lines of Ca 317.9 nm, Fe 238.2 nm, Mn 257.6 nm, Mg 285.2 nm, P 213.6 nm, Zn206.2 nm, Na 589.5 nm, and K 766.5 nm were also measured.

### 2.3 Statistical Analysis

All studies were performed in triplicate. The results are expressed as the mean ± standard deviation (SD). Data were analyzed using one-way analysis of variance (ANOVA), and differences between means were determined using Tukey’s honestly significant difference (HSD) test at *p* < 0.05. SPSS 22.0 software (SPSS Inc., Chicago, IL) was used for statistical analysis. SigmaPlot 12.0 statistical analysis software (Systat Software, Inc., San Jose, California) generated three-dimensional RSM plots. MLR and PSO were performed using the Spyder IDE (version 5.4.3) in Anaconda Software (Anaconda, Inc., Austin, Texas) and the Python programming language (version 3.9).

## 3 Results and Discussion

### 3.1 Multiple Linear Regression (MLR) and Response Surface Methodology (RSM)

#### 3.1.1 Multiple Linear Regression (MLR) Modeling

The MLR equation expresses the linear relationship between the dependent and three independent variables. It was used to understand the effects of thermosonication on chlorophyll and ascorbic acid in the parsley juice. Second-order regression models were constructed using data from 20 experimental runs obtained from a 3-factorial central composite design. The model for total chlorophyll (mg/100 mL) is given in eq 14, and the model for AA (mg/100 g) is given in eq 15.
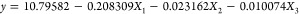
14
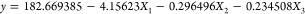
15

#### 3.1.2 Response Surface Methodology (RSM)

The experimental and predicted responses of thermosonication treatment by RSM have been determined. RSM modeling was conducted to assess the effects of independent variables on total chlorophyll and ascorbic acid, and optimal values were identified. Variance analysis (ANOVA) was utilized to determine the statistical significance of the model (*p* < 0.05). Lack-of-fit tests, *R*^2^, and adjusted *R*^2^ coefficients, along with ANOVA results, were evaluated as fitness indicators of the model. Independent variables were determined within the range of time (*X*_1_), amplitude (*X*_2_), and temperature (*X*_3_). Thermosonication parameters were optimized by using a numerical optimization approach. The analysis of variance (ANOVA) for parsley juice samples regarding total chlorophyll and ascorbic acid is highly significant, with a high coefficient of determination (*R*^2^) for the model (*p* < 0.05). This result indicates a high correlation between the experimental and predicted data for parsley juice’s total chlorophyll and ascorbic acid values. According to the analysis of variance results, the linear effects of parameters *X*_1_ and *X*_2_ on chlorophyll response are significant (*p* < 0.05). In contrast, the linear effect of *X*_3_ on the chlorophyll response is not significant (*p* > 0.05). On the response value of ascorbic acid, the effects of all three parameters are significant (*p* < 0.05). This indicates that the thermosonication parameters highly influence ascorbic acid. Baltacıoglu et al. applied thermosonication treatment to apple juice at different temperatures, amplitudes, and durations. The processing parameters that best preserved total phenolic content and antioxidant activity were determined to be 80% amplitude, 60 °C temperature, and 15 min duration. It was concluded that thermosonication preserves bioactive components.^33^ In a study similar to ours, conducted by Dündar et al., it was found that thermosonication treatment influenced the ascorbic acid content in strawberry nectar production.^34^ This phenomenon was explained by eliminating dissolved oxygen after sonication.^35^ The ANOVA results for parsley juice thermosonication are presented in Table 2.

**Table 2:** ANOVA Results of Regression Coefficients Obtained by RSM of Total Chlorophyll and Ascorbic Acid Responses as a Result of Thermosonication^a^

		**total chlorophyll(mg/100 mL)**				**ascorbic acid(mg/100 g)**			
**source**	**DF**	**adj SS**	**adj MS**	***F*-value**	***P*-value**	**adj SS**	**adj MS**	***F*-value**	***P*-value**
**model**	9	14.94	1.66	170.55	0.000	2532.77	281.42	114.56	0.000
**linear**	3	3.44	1.15	117.71	0.000	1273.68	424.56	172.83	0.000
***X*_1_**	1	2.63	2.63	270.55	0.000	1110.39	1110.39	452.02	0.000
***X*_2_**	1	0.78	0.78	80.04	0.000	141.79	141.79	57.72	0.000
***X*_3_**	1	0.02	0.02	2.55	0.141	21.51	21.51	8.75	0.014
**square**	3	8.20	2.73	280.97	0.000	1077.55	359.18	146.22	0.000
***X*_1_^2^**	1	4.16	4.16	427.66	0.000	195.35	195.35	79.52	0.000
***X*_2_^2^**	1	5.54	5.54	569.15	0.000	333.34	333.34	135.70	0.000
***X*_3_^2^**	1	0.13	0.13	12.87	0.005	345.86	345.86	140.79	0.000
**2-way interaction**	3	3.30	1.10	112.97	0.000	181.53	60.51	24.63	0.000
*X*_1_* *X*_2_	1	0.05	0.05	5.10	0.048	7.01	7.01	2.85	0.122
*X*_1_* *X*_3_	1	0.43	0.43	43.97	0.000	85.48	85.48	34.80	0.000
*X*_2_* *X*_3_	1	2.82	2.82	289.85	0.000	89.04	89.04	36.25	0.000
**error**	10	0.10	0.01			24.57	2.46		
**lack-of-fit**	5	0.04	0.01	0.85	0.570	20.24	4.05	4.68	0.058
**pure error**	5	0.05	0.01			4.33	0.87		
**total**	19	15.03				2557.34			
***R*^2^**		99.35%				99.04%			
**adj *R*^2^**		98.77%				98.17%			
**pred. *R*^2^**		97.26%				93.37%			

a *X*_1_: time; *X*_2_: amplitude; *X*_3_: temperature; DF: degrees of freedom; *R*^2^—coefficient of determination; AA: Ascorbic acid; *p* < 0.05, significant differences; *p* < 0.01, very significant differences.

**Figure 2:**
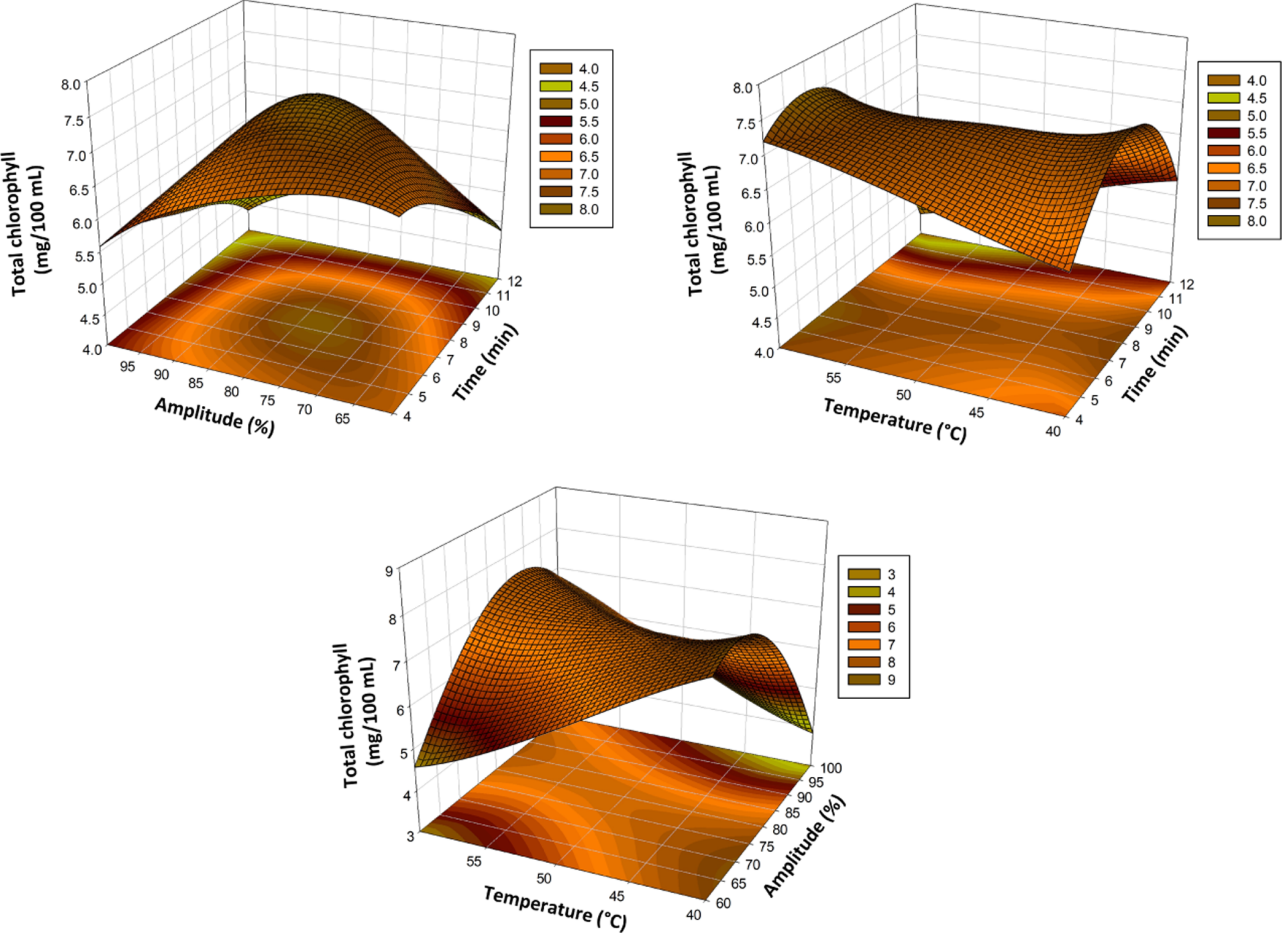
Response surface plots (3D) of total chlorophyll as functions of significant interaction factors.

Response surface plots (3-dimensional) were created to determine the optimum values for the independent variables total chlorophyll and ascorbic acid. The effect of thermosonication on chlorophyll is explained by the response surface plot shown in Figure 2. When the effects of time and amplitude were examined, a general increase in the level of chlorophyll was detected. The *R*^2^ value of the RSM modeling level showed a high fit of 99.35% (Table 2). Two-way and one-way effects of modeling were found to be statistically significant (*p* < 0.05).

**Figure 3:**
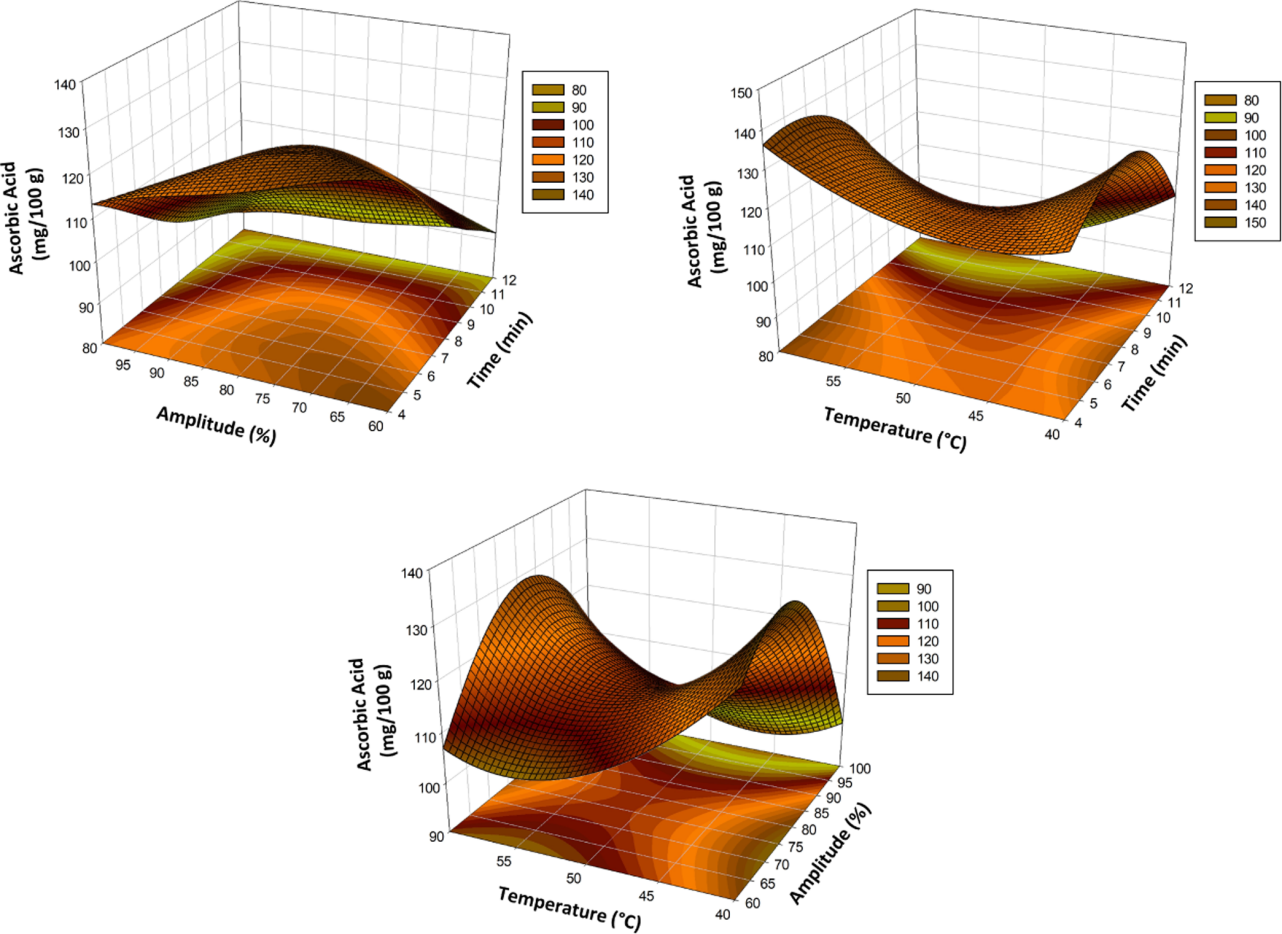
Response surface plots (3D) of ascorbic acid RSM as functions of significant interaction factors.

The effect of thermosonication on ascorbic acid is explained by the response surface graph shown in Figure 3. It was determined that the linear effects of time and temperature values on the ascorbic acid response were significant (*p* < 0.05). The *R*^2^ value of the RSM modeling level showed a high fit of 99.4% (Table 2).

#### 3.1.3 Comparison of RSM and MLR Models

**Figure 4:**
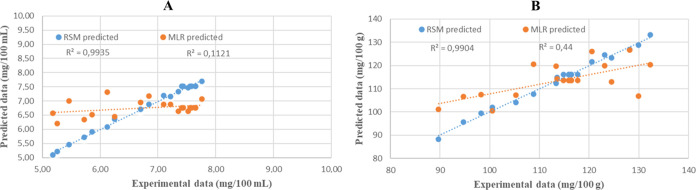
Parity plots.

Some studies in the literature show that RSM and MLR are used together for optimization.^36,37^ These two methods are adopted to analyze the experimental data and optimize the system parameters. Table 3 shows the performance metrics of RSM and MLR prediction models, and Figure 4 shows the regression plot of RSM and MLR. When the *R*^2^ metric of the models for total chlorophyll (mg/100 mL) and AA (mg/100 g) are analyzed in Table 3, it is seen that RSM shows a good fit as it is almost close to 1. The low RMSE metrics of the RSM models indicate that the RSM is a good fit.

Table 3 shows that the RSM technique is more suitable than the MLR technique for modeling total chlorophyll (mg/100 mL) and AA (mg/100 g). The MLR model usually requires more data to achieve better results. RSM, on the other hand, can achieve good results with less data. In addition, the relationship between dependent variables may not be linear. RSM is more successful in modeling nonlinear relationships than MLR.^37^ RSM can make more successful predictions than regression models.

**Table 3:** RSM and MLR Comparison^a^

	**total chlorophyll(mg/100 mL)**		**ascorbic acid(mg/100 g)**	
**parameters**	**RSM**	**MLR**	**RSM**	**MLR**
*R*^2^	0.99	0.11	0.99	0.44
RMSE	0.007	0.82	1.110	8.49
AAD (%)	1.16	15.15	1.220	7.45

a RSM stands for response surface methodology, MLR for multiple linear regression, RMSE for root-mean-square error, and ADD for average drop in performance.

The performance indicators *R*^2^, RMSE, and AAD (%) were used to test the significance of the models developed. The *R*^2^ value of the model for total chlorophyll (mg/100 mL) was 0.11, the RMSE value was 0.82, and the AAD (%) value was 15.15. The *R*^2^ value of the model for ascorbic acid (mg/100 g) was 0.44, the RMSE value was 8.49, and the ADD (%) value was 7.45.

While MLR models linear relationships between variables, RSM modeling considers more complex relationships (quadratic). Due to these characteristics, RSM can achieve higher *R*^2^ values as it can explain more variance. A low *R*^2^ value does not necessarily mean the model is invalid; in fact, *R*^2^ values, typically between 0.3 and 0.7 in our range, reflect the model’s context-specific applicability and usefulness. We also evaluate the performance of our model using other statistical measures, such as ADD and RMSE, which provide a more balanced picture of the model’s overall accuracy. In this context, the MLR model used was selected in accordance with our research questions and data structure.

### 3.2 Particle Swarm Algorithm (PSO)

The PSO algorithm was created, as shown in the flowchart in Figure 1. The fitness functions *f* were realized using the equations for total chlorophyll (mg 100 mL) and AA (mg 100 g) obtained from RSM and MLR. In eq 5, w *c*_1_ and *c*_2_ are non-negative real parameters.^38^ The inertia weight *w* is linearly decreased at each iteration, as given in eq 16. *k* is the number of iterations at that moment, *w*_min_ is the initial value of the inertia weight, *w*_max_ is the final value, and *k*_max_ is the maximum number of iterations.^39^*w* is the range of (0.1,1). *c*_1_ and *c*_2_ are the parameters of the learning factor. *c*_1_ is the cognitive coefficient, and *c*_2_ is the social coefficient. It controls the relative influence between the particle’s emory and that of the swarm.^38^ In this study, the parameters *c*_1_ and *c*_2_ were set to 0.5, and the number of iterations was set to 50. Although a large number of particles increase the diversity and exploration ability of the swarm, it also leads to difficulties in the calculation. It is known that PSO does not respond to population size if the number of particles is more than 50.^40^ Therefore, the number of particles was set to 50. The fitness functions include three independent variables. The lower and upper limits of the independent variables are given in Table 4.
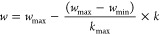
16

**Table 4:** Fitness Function’s Lower Limit and Upper Limit Values^a^

	**lower limit**	**upper limit**
***X***_**1**_	4	12
***X*_2_**	60	100
***X***_**3**_	40	60

a *X*_1_: time; *X*_2_: amplitude; *X*_3_: temperature.

With PSO, models can approach specific optimization goals by randomly navigating multidimensional parameter spaces. Table 5 shows the optimal values from PSO optimization of the equations for total chlorophyll (100 mg) and AA (100 mg) from RSM and MLR.

**Table 5:** Optimization Results in PSO^a^

	**total chlorophyll(mg 100 mL)**		**AA(mg 100 g)**	
	**RSM**	**MLR**	**RSM**	**MLR**
***X***_**1**_	7.96	4.10	4.04	7.61
***X***_**2**_	64.52	61.88	83.25	60.18
***X***_**3**_	40.00	42.59	60.00	40.78
**best solution**	8.38	8.08	147.46	154.91

a *X*_1_: time; *X*_2_: amplitude; *X*_3_: temperature.

**Figure 5:**
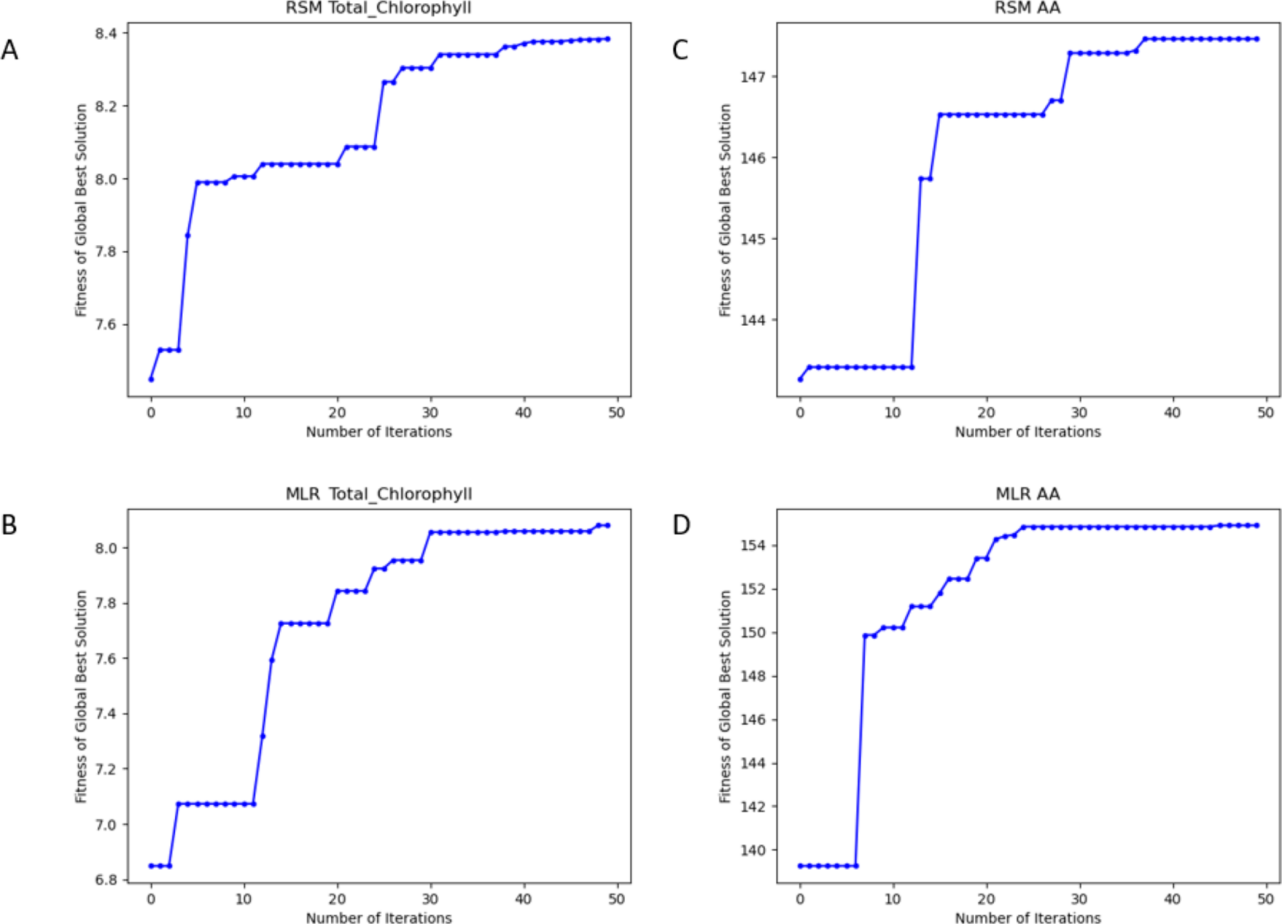
(A–D). Change of fitness value of total chlorophyll (mg 100 mL) and AA (mg 100 g) equations obtained from RSM and MLR according to the number of generations. (A) Total chlorophyll–RSM, (B) MLR–Total chlorophyll, (C) RSM–AA, (D) MLR–AA.

Figure 5A–D shows the variation of the fitness function value of the equations according to the number of generations. For all models, PSO shows fast convergence in the first few iterations. The fastest optimal result is shown in Figure 5C. The speed at which PSO converges to optimal results is promising.

### 3.3 Total Bioactive Compounds and Radical Scavenging Abilities

**Figure 6:**
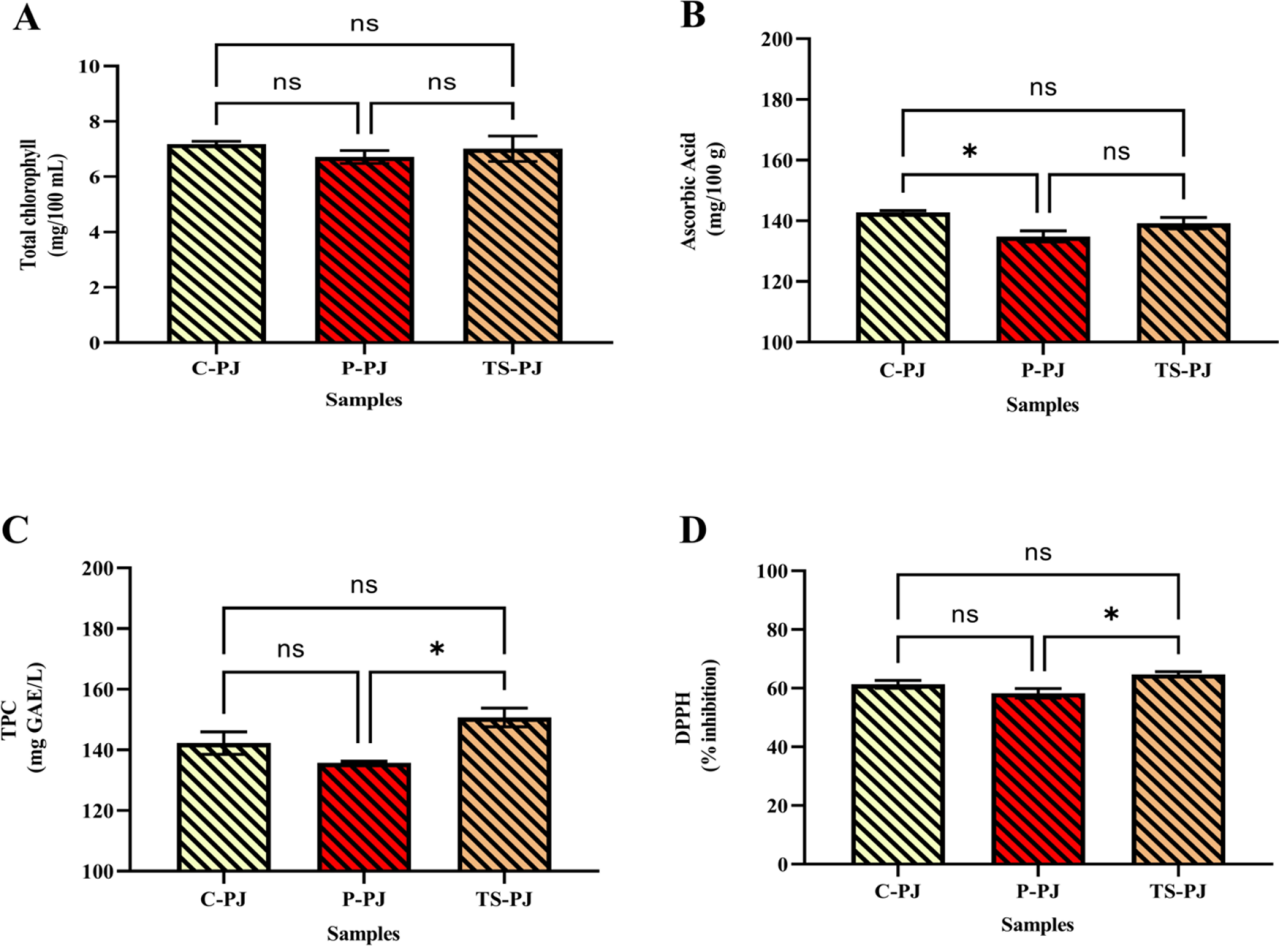
Total chlorophyll (A), ascorbic acid (B), TPC (C), and DPPH (D). The symbols at the top of the bars indicate statistically significant differences (*p < 0.05). C-PJ: Control parsley juice; P-PJ: pasteurized treated parsley juice; TS-PJ: thermosonication-treated parsley juice; ns: not significant.

Parsley is a rich source of phenolic compounds, ascorbic acid, flavonoids, and carotenoids.^41^ The results of the TPC, ascorbic acid, total chlorophyll, and DPPH tests on the parsley samples are shown in Figure 6. The TPC and DPPH values in the C-PJ, P-PJ, and TS-PJ samples were 142.27 mg GAE/100 mL, 135.81 mg GAE/100 mL, and 150.73 mg GAE/100 mL; 61.27, 58.28, and 64.74% respectively. Although there was no significant difference between the control sample of parsley juice and the sample treated with ultrasound in TPC and DPPH values, a significant decrease was observed in the pasteurized sample. It is thought that the significant decrease in TPC and DPPH values of pasteurized parsley juices is due to the sensitivity of these components to the heat treatment applied. Similarly, Ertik et al. (2023) reported that parsley extract at concentrations of 500 and 750 μg/mL was a strong DPPH radical scavenger (46.65 ± 0.48 and 60.05 ± 0.37%, respectively).^41^ In another study, the amount of TPC in the parsley extract was found to be 40 mg per gram.^42^ Papuc et al. found that the TPC value was 14.87 ± 1.03 mg GAE/100 mL in parsley juice.^43^

It is thought that the higher TPC production in our study may be due to different production conditions and the difference in the region where parsley is obtained. Similar to our research, Tokatlı Demirok and Yıkmış (2022) found significantly higher TPC and DPPH values in the ultrasonically treated tangerine juice sample compared to the pasteurized sample.^44^ There was no significant difference in total chlorophyll and ascorbic acid content between C-PJ and TS-PJ samples (7.18 mg/100 mL–7.02 mg/100 mL and 142.83 mg/100 g–139.23 mg/100 g). This result shows that thermosonication application provides advantages in terms of ascorbic acid and total chlorophyll content compared with pasteurization application. Mierzwa and Szadzińska found that combining hot air, microwaves, and ultrasound significantly increased vitamin C levels.^45^ We may obtain different results from our study due to the synergistic effect of the combination of other treatments. Similar to the present study, another study found that the application of ultrasound did not cause a significant change in the chlorophyll content of parsley.^46^

### 3.4 Analysis of Phenolic Compounds

Phenolic compounds, which are intensely found in fruits and vegetables, significantly affect human health with their antioxidant properties. With their structure in the food matrix, they are essential compounds in terms of the quality and stability of the foods and the development of new products.^47^ The aim was to investigate the changes in phenolic compounds after processing parsley juice with thermal pasteurization and ultrasound. The results regarding the effects of treatment using ultrasound and thermal pasteurization on polyphenol contents are shown in Table 6.

**Table 6:** Properties of Phenolic Compounds of C-PJ, P-PJ, and TS-PJ Samples^a^

	**samples(μg/mL)**		
**compounds**	**C-PJ**	**P-PJ**	**TS-PJ**
gallic acid	39.03 ± 1.55^a^	37.14 ± 3.41^a^	51.51 ± 1.16^b^
hydroxybenzoic acid	1.68 ± 0.15^a^	1.32 ± 0.16^a^	1.11 ± 0.16^a^
vanillic acid	0.30 ± 0.04^a^	0.26 ± 0.11^a^	0.56 ± 0.06^a^
gentisic acid	0.27 ± 0.01^a^	0.25 ± 0.03^a^	0.37 ± 0.10^a^
p-coumaric acid	1.96 ± 0.02^a^	1.63 ± 0.08^a^	1.37 ± 0.25^a^
rutin	0.67 ± 0.03^a^	0.67 ± 0.09^a^	0.69 ± 0.07^a^
ferulic acid	0.21 ± 0.01^b^	0.12 ± 0.03^a^	0.18 ± 0.02^ab^
naringin	16.94 ± 0.54^ab^	11.27 ± 2.12^a^	20.65 ± 2.42^b^
*o*-coumaric acid	0.19 ± 0.02^a^	0.12 ± 0.02^a^	0.15 ± 0.01^a^
neohesperidin	n.d	1.69 ± 0.08^a^	2.01 ± 0.11^a^
resveratrol	0.12 ± 0.02^a^	0.11 ± 0.01^a^	0.13 ± 0.02^a^
quercetin	1.11 ± 0.09^a^	0.77 ± 0.06^a^	1.12 ± 0.18^a^
trans-cinnamic acid	2.00 ± 0.08^a^	2.21 ± 0.19^a^	2.16 ± 0.24^a^
alizarin	0.70 ± 0.04^a^	3.81 ± 0.15^b^	0.52 ± 0.05^a^
flavon	0.91 ± 0.07^a^	1.46 ± 0.18^b^	n.d.

a Results are presented as mean ± standard deviation (*n* = 3). Values with the different letters within the line are significantly different (*p* < 0.05). C-PJ: Control parsley juice; P-PJ: pasteurized treated parsley juice; TS-PJ: tThermosonication-treated parsley juice; n.d.: not detected.

The results showed that 15 polyphenols were detected in the C-PJ, P-PJ, and TS-PJ samples. Gallic acid was analyzed as the primary phenolic compound in parsley juice based on available standards. Also, naringin derivatives were identified in considerable amounts. There was a significant (*p* < 0.05) increase in ascorbic and gallic acid contents in ultrasound-treated TS-PJ. Ultrasound applications create microbubbles (cavitation bubbles) in sudden and high-pressure changes in plant tissue cells with cavitation caused by low-frequency sound waves. These bubbles burst, breaking down cell membranes, and secondary metabolites (i.e., carotenoids, lycopene, phenolics) are released, increasing the sample’s polyphenols.^48−50^ The increase in the concentrations of vanillic acid, gentisic acid, rutin, quercitin, and trans-cinnamic acid by the investigations was reported by Erdal et al., who found a similar increased influence was ultrasound-treated gilaburu vinegar samples.^505^ Also, an insignificant increase in these phenolic compounds after ultrasound treatment confirms the research of Kidoń and Narasimhan.^51^ The studies conducted reported that the phenolic content in samples such as lettuce (*Lactuca sativa*),^52^ strawberry,^53^ and strawberry juice^54^ increased after ultrasound application at certain temperatures and time intervals according to the method of ultrasound.

### 3.5 Analysis of Minerals

**Figure 7:**
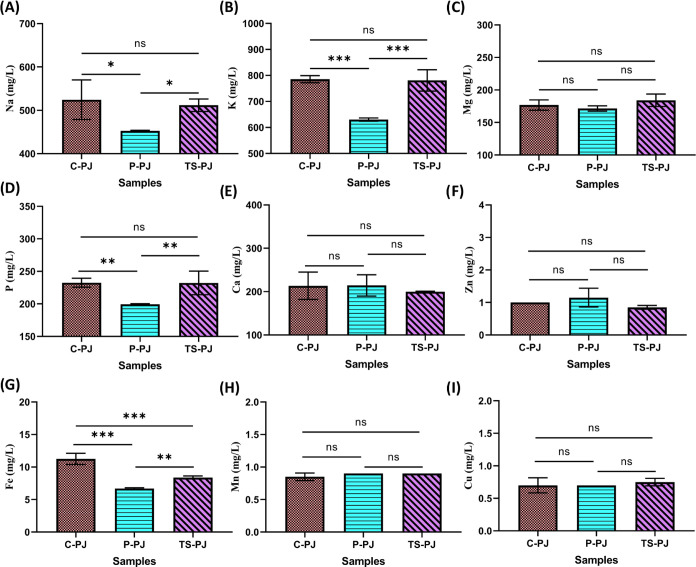
Results for Na (A), K (B), Mg (C), P (D), Ca (E), Zn (F), Fe (G), Mn (H), and Cu (I) minerals of C-PJ, P-PJ, and TS-PJ samples. Letters at the top of the bars indicate statistically significant differences (*n* = 3 ± SD). C-PJ: Control parsley juice; P-PJ: pasteurized treated parsley juice; TS-PJ: thermosonication-treated parsley juice; ns: not significant.

Minerals are essential ingredients in our food. They can build materials for our bones, influence nerve and muscle function, and regulate the body’s water balance.^55^ Vegetables, an essential part of the human diet, help us get vitamins and minerals, vital nutrients in living.^56^ The mineral results of the parsley juice samples are shown in Figure 7. The results show that 9 minerals were detected in the C-PJ, P-P, and UT-PJ samples. The highest mineral contents in C-PJ vegetable juice samples were K (785.70 mg/L), Na (524.45 mg/L), P (232.55 mg/L), Ca (213.45 mg/L), Mg (176.85), Fe (11.25 mg/L), Zn (1 mg/L), and Mn (0.85 mg/L), and the lowest mineral content was found as Cu (0.05 mg), respectively. However, no significant difference was observed in Na, K, P, Ca, and Zn content between the Ultrasound-treated samples and the control, which could be attributed to the low level in parsley juice except Fe. Mineral contents of control parsley samples (C-PJ) showed a decrease in Na, K, P, and Fe contents in pasteurized treated samples (P-PJ) (*p* < 0.05). In the studies, Na and K from macro minerals and Fe and Zn from micro minerals were found to be high in vegetable juices.^57^ Similar results were also found in our study. Also, in other studies, the mineral content of wheatgrass juice K (3383.4 mg/L) and P (1216.9 mg/L) contents were found to be high, but the Mg (133.9 mg/L) and Ca (39.2 mg/L) contents were low compared to our study results.^58,59^

Thermal technologies with heat treatment soften the steam food matrix in vegetable products and improve their bioavailability. However, heat treatment and the parameters used can negatively affect the structure of the food matrix.^60^ Mehmood and Zeb stated that the steaming method preserves the vegetable content and vitamin and mineral values better, while other methods, in which they tried frying, microwaving, boiling, and steamed methods, reduce the nutritional content of leafy vegetables, such as broccoli and spinach.^61^

Using nonthermal technologies increases the shelf life of fruit and vegetable juices and ensures that they are presented as microbiologically safe food. This method has been reported to increase processed beverages’ nutritional and functional benefits, improve quality parameters, break the cell wall with cavitational effects, and inactivate microorganisms and enzymes.^62^ Therefore, ultrasound technology uses fresh tomato juice,^63^ spinach juice,^64,65^ pumpkin juice,^66^ wheatgrass juice,^59^ and kutkura (Meyna spinosa) juice.^67^ As there is limited information relating to the mineral content of parsley juice and its high variability is dependent on different conditions, it is not easy to compare the mineral composition with other juices. The concentration of mineral substances that have significant health effects in vegetable juice varies depending on factors such as growing conditions of vegetables, fertilizer use, soil composition, planting and harvesting time, harvesting processing conditions, and storage and temperature.^59,68^ A vast amount of loss of nutrients is seen in vitamins and minerals during the processing of fruits and vegetables. For this reason, the research focused on the effect of ultrasound technology, one of the green technologies, on the mineral content of vegetable juice. The absence of any studies in the literature, as mentioned in the article, regarding the observation of changes in mineral substances in parsley juice through the application of different methods is also an important issue.

## 4 Conclusions

Parsley juice is an important healthy fruit juice due to its bioactive components and nutritional content. Also, it has a good effect on curing many health disorders. In this study, parsley juice was applied to thermosonication treatments, and as a result of RSM optimization, it was enriched in terms of phenolics, flavonoids, total antioxidant compounds, and mineral contents. Bioactive components of parsley juice were increased by the thermosonication processing. It was determined that thermosonication preserves the antioxidant, phenolic, and mineral content better than thermal pasteurization. C-PJ was detected to contain nine different mineral elements (Na, K, Mg, P, Ca, Fe, Mn, and Cu). Thermosonication caused an increase in K mineral. Researchers used the response surface methodology (RSM) to describe how ultrasound technology affected the properties of parsley juice. They compared the RSM method with multiple linear regression (MLR) and found that RSM was better at modeling nonlinear relationships and in studies with few experiments. Particle swarm optimization (PSO) was used to find the best values for the equations obtained with RSM and MLR. By using a combination of these methods, researchers were able to model complex relationships in the data and find optimal values. However, further studies are needed to improve specific analyses such as sensory properties and shelf life of thermosonication-treated parsley juice. Also, in the following research, interpreting anticancer, antimicrobial, and bioavailability properties is suggested. These results implied that the thermosensation process might potentially replace the traditional thermal processing of liquid products.
